# Active Polysaccharide-Based Films Incorporated with Essential Oils for Extending the Shelf Life of Sliced Soft Bread

**DOI:** 10.3390/molecules29194664

**Published:** 2024-09-30

**Authors:** Nooshin Noshirvani, Cédric Le Coz, Christian Gardrat, Babak Ghanbarzadeh, Véronique Coma

**Affiliations:** 1 Laboratoire de Chimie des Polymères Organiques, Université de Bordeaux, CNRS, Bordeaux INP, UMR 5629, 16 Avenue Pey-Berland, F-33600 Pessac, France; n.noshirvani@basu.ac.ir (N.N.); cedric.lecoz@enscbp.fr (C.L.C.); christian.gardrat@u-bordeaux.fr (C.G.); 2 Department of Food Science and Technology, Tuyserkan Faculty of Engineering & Natural Resources, Bu-Ali Sina University, Hamedan 65178-38695, Iran; 3 Department of Food Science and Technology, Faculty of Agriculture, University of Tabriz, Tabriz P.O. Box 51666-16471, Iran; ghanbarzadeh@tabrizu.ac.ir

**Keywords:** chitosan, carboxymethylcellulose, oleic acid, antifungal, antioxidant, cinnamon essential oil, ginger essential oil, bread preservation

## Abstract

Active, fully biobased film-forming dispersions (FFDs) with highly promising results for sliced soft bread preservation were successfully elaborated from carboxymethyl cellulose (CMC) and chitosan (CH) using a simple method based on pH adjustments. They consisted of the association of polysaccharides and oleic acid (OL) added with cinnamon (CEO) or ginger (GEO) essential oils. The chemical compositions of the commercial essential oils were first determined via GC/MS, with less than 3% of compounds unidentified. The films obtained from FFDs were characterized by SEM, FTIR and DSC, indicating specific microstructures and some interactions between essential oils and the polymer matrix. CEO-based films exhibited higher antioxidant properties and a lower minimal inhibitory concentration in terms of antifungal properties. From experiments on sliced soft bread, the ginger-based films could increase the shelf life up to 20 days longer than that of the control. Even more promising, cinnamon-based films led to complete fungal inhibition in bread slices that was maintained beyond 60 days. Enumeration of the yeasts and molds for the FFD-coated breads revealed complete inhibition even after 15 days of storage with the FFDs containing the highest concentration of CEO.

## 1. Introduction

Bread in its various forms is regarded as one of the most popular foods all over the world [1]. For centuries, it has constituted the dominant part of people’s diets and supplied the major proportion of energy and nutrient needs for the human body (carbohydrates, proteins, dietary fibers, minerals and vitamins). Bread shelf life, in particular for soft bread, is generally restricted to a few days [2] by two main factors: (i) complex physical changes known as staling and (ii) fungal development, which leads to spoilage, loss of texture and flavor, off-flavor development and the production of mycotoxins that are potentially harmful to human health [3,4]. The *Penicillium*, *Aspergillus* and *Fusarium* species are by far the most common fungi polluting wheat, rye and corn grains [5,6]. Up to now, the challenge for bakers has been to extend the shelf life while reducing the quantities of additives in bread products. Plenty of methods have been reviewed recently [7,8]. The increase in consumers’ refusal of non-natural products as well as the environmental pollution caused by synthetic polymers have oriented the active packaging research towards the use of compounds from nature such as essential oils (EOs) and biopolymers [9,10].

Essential oils are aromatic liquid oils that have been known as good antimicrobial and antioxidant agents since ancient times [11]; cinnamon and ginger EOs, generally recognized as safe (GRAS), are compatible with foods, particularly with bakery products [12,13]. Among biopolymers, chitosan (CH) is a linear polysaccharide composed of β-(1→4)-linked 2-acetamido-2-deoxyglucopyranose and 2-amino-deoxyglucopyranose obtained by partial deacetylation of natural chitin; it is non-toxic, filmogenic and biodegradable. In acidic conditions (pH < 6.5), it has antimicrobial and antioxidant properties, and it has high potential in food packaging application [14]. Blending CMC with chitosan enhances the barrier and mechanical properties of pure films; however, the presence of hydroxyl groups makes them sensitive to water [15]. To address this issue, adding hydrophobic compounds such as fatty acids can help reduce the hydrophilicity [16]. Previous studies have shown a reduction in the water vapor permeability of chitosan films incorporated with sunflower oil [17], olive oil [18] and oleic acid (OL) [19]; the same result was obtained for CMC films incorporated with oleic acid [20].

This paper is devoted to (i) determining the composition of the selected commercially available cinnamon essential oil (CEO) and ginger essential oil (GEO), (ii) developing stable film-forming dispersions (FFDs) using CMC, CH and OL with or without EOs, (iii) the study of films prepared from FFDs, (iv) the investigation of the antifungal properties of films against the growth of *Penicillium digitatum*, a strain particularly detrimental to bread and, finally, (v) a study of the impact of the biobased materials (films and coating) on the microbial contamination and shelf life of sliced soft bread.

## 2. Results and Discussion

### 2.1. Composition of Ginger and Cinnamon Essential Oils

According to GC/MS analyses, unidentified compounds represented less than 3% of the total ion chromatogram area for both essential oils. GEO contained α-pinene (1.1%), camphene (3.3%), eucalyptol (1.3%), *ar*-curcumene (13.9%), α-zingiberene (48.2%), α-farnesene (1.3%), β-bisabolene (12.6%) and β-sesquiphellandrene (15.4%). These results have a good qualitative correlation with a recent study [21]. CEO contained mainly *trans*-cinnamaldehyde, as indicated by the supplier (91.6%), and small amounts of several other products identified as benzaldehyde (0.8%), dihydrocinnamaldehyde (0.4%), phenylethanol (0.4%), cinnamyl acetate (3.6%), *o*-methoxycinnamaldehyde (0.6%) and coumarine (1.2%), which have already been observed in *Cinnamomum cassia* essential oil [22,23,24].

### 2.2. Elaboration and Characterization of CMC-Chitosan-Oleic Acid-Essential Oil (CMC-CH-OL-EO) Films

#### 2.2.1. Preparation of Film-Forming Dispersions (FFDs)

**Figure 1:**
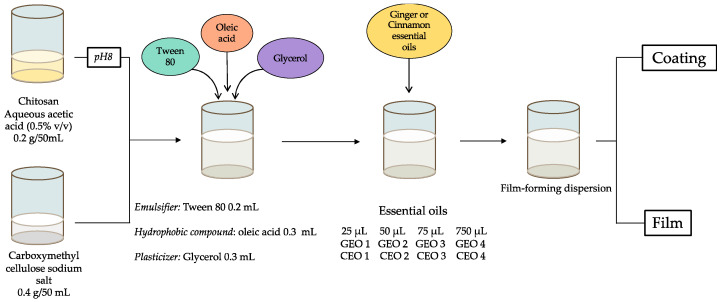
Experimental procedure to obtain stable film forming dispersions (FFDs) from chitosan and CMC biopolymers.

Preliminary studies were carried out to achieve a suitable formulation due to the anionic character of CMC (sodium salt) in aqueous solutions and the cationic character of chitosan in acidic solutions. By first adjusting the pH of the chitosan solution and the order of addition of the various components, a stable formulation was developed, preventing the formation of a tight gel. By modifying the CMC:chitosan weight ratio and the pH of the chitosan solution before mixing the two solutions, a convenient procedure was finally selected. A CMC:chitosan ratio of 2:1 (*w*/*w*) and an adjustment of the pH of the chitosan solution to 8.0 before mixing the two solutions were selected, as shown in Figure 1 and described in detail in the experimental section.

FFDs were used as a coating for sliced soft bread or to prepare films after solvent removal. It should be noted that, under these experimental conditions, chitosan was in its neutral form (NH_2_) and CMC and oleic acid were in their carboxylate form (CO_2_^−^).

In this paper, “CMC-CH-OL” corresponds to a material composed of CMC, CH, OL, Tween 80 and glycerol without any EOs. “CMC-CH-OL-CEO 1” is a material prepared from CMC, CH, OL, Tween 80, glycerol and CEO (25 µL).

#### 2.2.2. ATR-FTIR Spectra of Films

**Figure 2:**
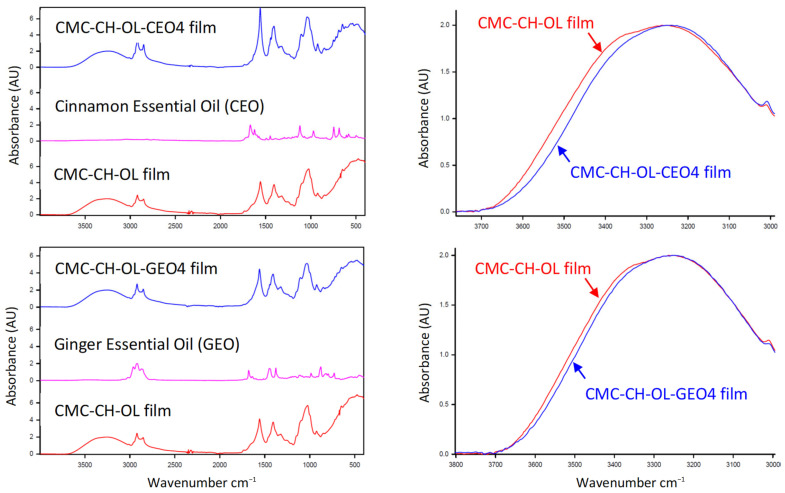
ATR-FTIR spectra of CMC-CH-OL-EO films (**left**) and zoom of the 3000–3800 cm^−1^ zone for the cinnamon (**right above**) and the ginger (**right below**) essential oils.

CMC-CH-OL films (control films) and those containing cinnamon and ginger essential oils at the highest concentrations (CEO 4 and GEO 4) were analyzed by ATR-FTIR (Figure 2) to study some potential interactions between the polysaccharide matrix and the essential oils.

For the *Cinnamomum cassia* essential oil, the major compound trans-cinnamaldehyde showed bands at 1670 and 1624 cm^−1^ corresponding to the characteristic stretching vibrations of an aldehyde function conjugated to a double bond [25]. For the *Zingiber officinale* Roscoe essential oil, the four sesquiterpenic hydrocarbons identified (α-zingiberene, β-sesquiphellandrene, *ar-*curcumene and β-bisabolene) account for the large part of the oil (~92%) and have similar structures (mixture of simple and double bonds). The infrared spectrum of the essential oil was compatible with their individual spectra, especially between 1350 and 1700 cm^−1^ [26]. The Tween 80 and oleic acid used in the preparation of the films are known to possess, in particular, a carbonyl double bond at 1736 cm^−1^ for the former and at 1707 cm^−1^ for the latter [27].

Several observations can be made from the spectra of CMC-CH-OL films: (i) two broad bands appeared between 3100 and 3600 cm^−1^ due to partially overlapped ν_O-H_ and ν_N-H_ stretching vibrations in the polysaccharide matrix; (ii) oleic acid gave clear ν_C-H_ stretchings at around 2920 and 2855 cm^−1^; (iii) the ν_C=O_ bond of Tween 80 appeared at 1736 cm^−1^; (iv) the bands at 1560 and 1410 cm^−1^ belonged to the CMC-CH matrix [27]; (v) the shoulder appearing at about 1650 cm^−1^ could be attributed to the chitosan amide I vibration; (vi) the broadening of the band centered at 1560 cm^−1^ could be explained by the presence of carboxylate ions due to the pH increasing [27]; and (vii) the complex bands between 900 and 1200 cm^−1^ might be attributed essentially to the carbohydrate skeleton.

Significant changes can be observed in the infrared spectra of CMC-CH-OL films loaded with CEO and GEO, particularly in the 3100–3600 cm^−1^ zone (Figure 2). The ν_O-H_ stretching bands were shifted to lower wavenumbers, from ~3345 cm^−1^ to ~3254 cm^−1^ for CEO and ~3261 cm^−1^ for GEO, which indicated a change in the matrix structure. The bands at 1736, 1560 and 1410 cm^−1^, which belonged, respectively, to Tween 80 and the CMC-CH matrix, remained at the same wavelengths. It can be concluded that Tween 80 had no impact on the structure of the films. Furthermore, a distinction was observed in the CEO-loaded film, where two shoulders appeared around 1663 and 1636 cm^−1^. It appeared that upon incorporating CEO into the CMC-CH-OL films, the characteristic bands of cinnamaldehyde emerged in this infrared region with a significant shift. Such phenomena were not evident in the GEO-incorporated films.

As a result, the incorporation of essential oils created some modifications in the structure of the carbohydrate matrix. The presence of CEO or GEO partially reduced the initial network, probably by destroying the hydrogen bonds between the polymer chains, allowing the dissemination of the essential oils. In addition, the composition of the oils causes different behaviors. With GEO, which primarily consists of unsaturated hydrocarbons, no other changes seem to have occurred. Conversely, with CEO, which contains virtually only cinnamaldehyde, it seems that this molecule interfered with the matrix, perhaps through Van der Waals interactions linked to the molecule’s high dipole moment [28].

#### 2.2.3. Microstructures of the Films

**Figure 3:**
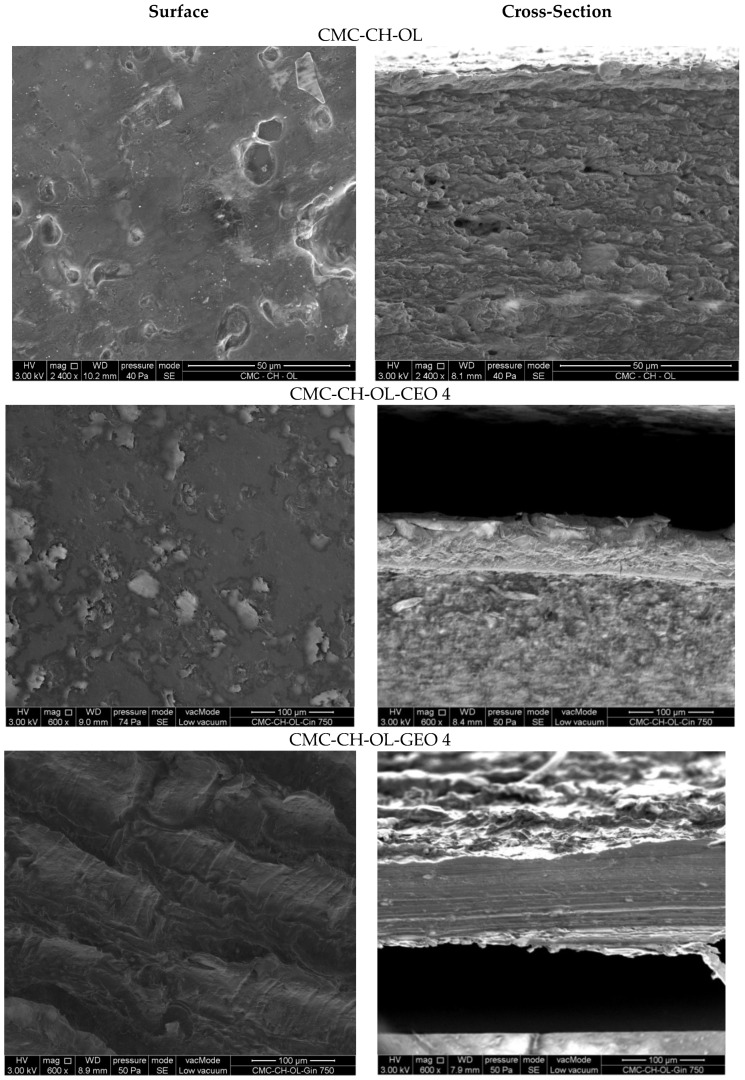
Surfaces (**left**) and cross sections (**right**) of CMC-CH-OL film, CMC-CH-OL-CEO 4 film and CMC-CH-OL-GEO 4 film.

SEM microstructures of the surfaces and cross sections of the CMC-CH-OL films with and without the selected essential oils are presented in Figure 3.

CMC-CH-OL film showed some big droplets, attributed to oleic acid not being well distributed in the continuous polymer matrix; some holes or cracks can also be observed on the cross section. The addition of CEO or GEO led to different film morphologies depending on the chemical compositions of the oils, as already described in a previous study with lower EO concentrations [29]. The largest differences were observed for the highest concentrations. In films incorporated with CEO 4, clearer zones can be observed on the film surface that are distributed non-uniformly. In films incorporated with GEO 4, the morphology is quite different, with some regular folds in the structure producing “hollows and bumps”, indicating different interactions between the matrix and GEO. There is a change in morphology as the amount of GEO increases [29]. In addition, the cross sections of the films were also different, although they remain compact and pore-free for both EOs. For films with GEO 4, the cross section is fairly regular, but for films with CEO 4, different phases appeared.

#### 2.2.4. Thermal Properties

##### Differential Scanning Calorimetry (DSC)

Differential scanning calorimetry (DSC) was used to investigate the possible interactions between EOs and the CMC-CH-OL matrix in the films. The results corresponding to the highest quantities of EOs (i.e., CEO 4 and GEO 4) are presented in Figure S1. As already mentioned (Section 2.1), analysis of the EOs via GC/MS indicated the presence of a major compound in the CEO (*trans*-cinnamaldehyde) and a large number of compounds in the GEO (mainly α-zingiberene, β-sesquiphellandrene, *ar*-curcumene, β-bisabolene). As a result, the thermal effects of the evaporation of each component will overlap with each other, leading to broad endothermic events on DSC curves, particularly with GEO. These peaks appeared at 159 °C for CEO and 138 °C for GEO, with ΔHs equal to 159 J/g and 72 J/g, respectively. After addition of EOs in CMC-CH-OL matrix, these peaks disappeared, which seemed to be in favor of interactions between the EOs and the matrix. The same type of phenomenon has already been observed for chitosan films with added naringin [30].

The interactions between polymers and EOs might allow the use of lower essential oil concentrations compared to direct addition due to the prolonged release of active compounds and thus higher surface concentration, as well as maintaining the presence of essential oils in the headspace of the packaging [31]. However, even though the decomposition temperature is above 180 °C for both oils, caution seems necessary when using these essential oils in a high-temperature environment [32].

##### Thermogravimetric Analysis (TGA) of Essential Oils 

The thermal behavior of essential oils is an important factor in their use in food applications, particularly their evaporation rates. By studying their mass loss versus time and/or temperature, TGA evaluates the thermal stability of EOs and estimates the activation energy due to their evaporation. The TGA curves obtained for four different heating rates are shown in Figure S2. For CEO, an abrupt profile was observed, indicating that there was only one event leading to complete degradation of the essential oil at 180 °C. This behavior was attributed to a zero-order vaporization process [33,34]. For GEO, the TGA curve was more complex with several events, but, similarly to CEO, it began under 200 °C.

The results of TGA can be used to evaluate activation energies according to two model-free methods described in Supplementary Part A, the Flynn–Wall–Ozawa (FWO) and Kissinger–Akahira–Sunose (KAS) methods. By plotting ln β versus 1/T_max_ and ln(β/T_max_^2^) versus 1/T_max_, for the FWO and KAS models, respectively, where β is the heating rate and T_max_ the temperature at which the vaporization rate is highest, a straight line was obtained using the least squares method. The slope values were used to calculate the activation energies and their standard deviations (Supplementary Part A). The E_a_ value obtained in this work for CEO concurs with the one (51 KJ/mol) given by Hazra et al. [34]. For GEO, no evaluation of the E_a_ seems to have been described previously.

#### 2.2.5. Total Phenolic Content and Antioxidant Activity of Films

The total phenolic content of the films (TPC) and DPPH scavenging methods were used to measure the antioxidant activity of the films incorporated or not with CEO or GEO.

TPC was measured using Folin–Ciocalteu reagent. In this method, the antioxidant is oxidized in an alkaline medium formed by a mixture of tungstate and molybdate ions, producing colored MoO^4+^ ions (λ = 760 nm) [35]. Gallic acid is commonly used as a reference standard.

The CMC-CH-OL films exhibited a low value of 1.75 mg gallic acid equivalent (GAE) per g of film (Figure S3A), which can be compared to the literature-reported values for chitosan films, specifically 2.38 mg GAE/g [36] and 1.95 mg GAE/g [37]. The authors indicated that this could be due to the formation of chromogens between the Folin-Ciocalteu reagent and non-phenolic compounds. In fact, the Folin-Ciocalteu method does not allow a specific determination of phenolic compounds, as it reacts with many non-phenolic compounds that are easily oxidized [38]. For instance, a TPC of approximately 1.4 mg GAE per mL of *Zingiber capitatum* Roxb essential oil has been reported without phenolics in the chemical composition of the oil [39]. The reagent does not solely measure phenols but reacts with any reducing substance. Hence, it quantifies the total reducing capacity of a sample, encompassing more than just phenolic compounds.

The addition of essential oils to the films resulted in an increase in TPC values as the oil concentration increased (Figure S3A). However, this increase was not proportional to the amount of essential oil added, and films incorporating CEO always showed higher values compared to those with GEO. It can be inferred that essential oils, which typically contain easily oxidizable compounds like terpenoids, yield positive results with the Folin–Ciocalteu reagent even without phenolic components in their composition. The discoloration reaction with molybdate ions appears complex, as the TPC values did not scale linearly with the amount of CEO and GEO incorporated into the films. These findings suggest potential interactions between the components of essential oils.

The DPPH test is based on a simple process: a molecule with a weak C-H bond can react with the colored (λ = 517 nm) and persistent 2,2-diphenyl-1-picrylhydrazyl radical (DPPH) to produce a colorless solution after abstraction of a hydrogen atom (DPPH-H). Results are often given as DPPH scavenging activity (%) but without waiting for the reaction to complete. The results are therefore particularly dependent on the reaction time and the DPPH concentration. The data can be compared only if the reaction conditions are exactly the same.

The antioxidant activity from DPPH analyses of films incorporated or not with CEO or GEO is presented in Figure S3B. The CMC-CH-OL films showed a DPPH scavenging effect of nearly 12%, which can be attributed to chitosan. It is known that chitosan is not an efficient scavenger of DPPH radicals [37,40,41]. The low activity is probably due to the reactions of free radicals with the amino groups of chitosan [37]. After incorporation of the essential oils into the films, the DPPH scavenging activity increased significantly (*p* < 0.05). It also increased as the concentration of EO increased, but not linearly, and CEO always showed higher scavenging activity than GEO. This greater antioxidant capacity of CEO compared to GEO has already been described [42]. At the highest concentrations of the EOs, the antioxidant activity was significantly improved (*p* < 0.05) compared to CMC-CH-OL films, by a factor of 2 and about 5.5 times after incorporation of GEO and CEO, respectively. The antioxidant activity of EOs depends on the nature and quantity of their components [43,44].

Both EOs do not contain phenolic compounds, which are generally known to have high antioxidant activities [45]. Some articles have provided an interesting answer regarding the origin of the antioxidant properties of certain individual compounds such as limonene (model for cyclic monoterpenes), γ-terpinene (model for terpenoids containing cyclohexadiene structures), linalool and citral (models for terpenoid alcohols and aldehydes) often present in essential oils [46,47]. The antioxidant behavior of these types of compounds is due to their higher rate of self-termination and cross-termination relative to the oxidizable substrate; overall, chain termination is increased and autoxidation efficiency is reduced. However, their effectiveness is lower compared to phenols and does not depend linearly on their concentration. Additionally, their efficacy is associated with the rate of chain termination of the oxidizable substrate. These observations certainly reflect the behavior of other components present in EOs and it is likely that interactions between all the components have synergistic or antagonistic effects, which are difficult to predict.

### 2.3. In Vitro Antifungal Activities of Essential Oils and Films

#### 2.3.1. Minimum Inhibitory Concentration (MIC) and Preliminary Antifungal Assays

The antifungal properties of the films were tested against the growth of *Penicillium digitatum* via the disc diffusion method. The results of the MIC for CEO and GEO against *P. digitatum* showed a lower MIC value for CEO (0.03 µL/mL) compared to GEO (4 µL/mL).

The antifungal properties of the films incorporated with CEO or GEO are presented in Table 1. The antifungal activity increased with increasing concentration of both EOs. However, CEO exhibited significantly (*p* < 0.05) stronger antifungal properties than GEO, in agreement with the MIC values. As expected, the best efficacy was obtained with CEO 4, with over 80% inhibition after 5 days of incubation, followed by CEO 3 and GEO 4, with around 25% inhibition.

These results are in agreement with other studies on the antifungal activities of CEO or GEO against several *Aspergillus* and *Penicillium* strains [31,48,49]. The antifungal activities of EOs depend on their concentration, water solubility and lipophilic character [6] and are the result of several compounds, even minor ones, acting synergistically [23,50,51,52]. The hydrophobic nature of essential oils allows them to integrate into the lipids of microbial cell membranes and mitochondria, disrupting their structure and permeability. This disruption leads to the leakage of H^+^ and K^+^ cations and other cellular components, ultimately resulting in cell death [44,53]. The main components of EOs, cinnamaldehyde and zingiberene for CEO and GEO, respectively, are probably mainly responsible for the antifungal properties of essential oils [54,55].

#### 2.3.2. Antifungal Properties of Active Materials on Sliced Soft Bread

Two techniques were used to evaluate the antifungal properties on bread. The first was to assess the inhibition of naturally occurring yeasts and molds by FFD coatings to create full contact between the material and the selected food. These experiments were carried out to determine whether inhibition was possible under near-optimal conditions of contact and identify the inhibitory potential of FFDs formulations. The corresponding FFD-based films were then tested to evaluate their potential for improving the shelf lives of such food products.

##### Antifungal Properties of FFD Coatings on Bread Slices

Contamination by yeasts and molds increased during storage (Table 2), but the increase was lower in bread slices coated by FFDs compared to the control samples.

After 15 days of incubation, the best antifungal activity was obtained for a FFD with CEO 4, which showed no fungal growth; films incorporated with GEO at the same incorporation ratio were always less effective. These results are in agreement with MIC values and the disc diffusion test results against *P. digitatum*. Similarly, a reduction in the yeast and mold levels in bread stored in methylcellulose films combined with clove and oregano EOs has previously been observed [56]. However, Kechichian et al. [57] reported an increase in yeast and mold levels in bread slices wrapped in biodegradable films made from starch and cinnamon powder; according to these authors, the higher water activity (a_w_) of the bread compared to the active films was the likely cause of their observations. In this study, no significant differences in a_w_ were observed (Table S1).

##### Evaluation of Fungal Growth in Bread Slices Sandwiched between Active Films

**Figure 4:**
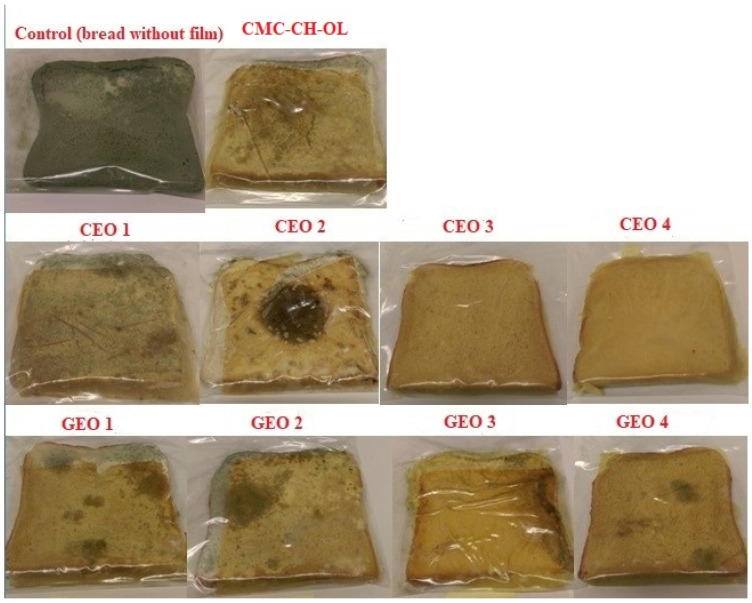
Visual appearance of bread slices packaged in polyethylene bags, sandwiched with different films after 8 weeks storage at 25 °C.

To study the effectiveness of active films on bread shelf life, bread slices were sandwiched between two pieces of film and incubated at 25 °C. Figure 4 shows the visual appearance of the bread slices wrapped in different films after 8 weeks of storage.

Bread slices wrapped in the control film showed earlier fungal development and after 7 days some fungal growth was observed (Table 3). A visual comparison of fungal growth indicated that the best results were obtained for films incorporated with the highest concentrations of cinnamon essential oil, CEO 3 and CEO 4 (Table 3 and Figure 4). Complete inhibition was observed until the end of the test (60 days).

Films incorporated with GEO showed lower antifungal properties than those with CEO, consistent with the MIC values against *P. digitatum*. However, a positive relationship was observed between the GEO concentration and antifungal activity, with the best results obtained at the highest concentration (GEO 4) after 28 days of storage. These findings confirm that incorporating CEO and GEO into CMC-CH-OL film delays fungal growth in bread. Similar results have been reported in the literature, where the shelf life of bread stored in gliadin films with 5% cinnamaldehyde increased to 27 days compared to fungal growth appearing after just 4 days at 25 °C without active films [58].

The shelf life, without mold growth, of garlic bread packaged in chitosan films increased from 7 days to 23 days when the films were incorporated with garlic oil [59]. An increase in the microbial shelf life of bread from 13 to 45 days has also been described for active polypropylene films incorporating 8% thymol and carvacrol [60]. According to these authors, carvacrol and thymol are gradually released into the headspace of bread during the shelf life. The gradual release of EOs from the polymer to the surface of bread is more effective than the direct addition of these compounds to the bread, since EO concentrations remain high on the bread surface throughout the shelf life. The formation of new interactions between the polymers and the CEO and GEO, confirmed by DSC and FTIR results, led to a reduction in the diffusion rates of the active compounds, enabling the antimicrobial properties to be retained for longer.

## 3. Materials and Methods

### 3.1. Materials

Chitosan (CH) (low molecular weight, 20–300 cP 1% *w*/*w* in acetic acid 1% *w*/*w* 25 °C, 50–190 kDa, deacetylation degree 75–85%) and sodium carboxymethyl cellulose (CMC) (high viscosity, 1500–3000 cP 1% in H_2_O 25 °C) were purchased from Sigma Aldrich (St. Louis, MO, USA). Analytical grade glycerol, oleic acid and Tween 80 were obtained from Merck (Darmstadt, Germany), DMSO from Scharlau Chemicals (Barcelona, Spain) and potato dextrose agar (PDA) from Biokar diagnostics (Beauvais, France). All other products were of analytical grade. Cinnamon and ginger essential oils (CEO and GEO) were obtained from a French local pharmacy: CEO was prepared from the leaves and young twigs of *Cinnamomum cassia* Nees ex-Blume and GEO from the rhizomes of *Zingiber officinale* Roscoe. Preservative-free sliced soft bread (Bio) was purchased from a local supermarket (Bordeaux, France). Its composition includes wheat flour (67%), water, sugar, rapeseed oil, natural flavor, salt, yeast, and wheat gluten.

### 3.2. Analysis of Commercial Essential Oils

Commercial essential oils were analyzed by GC/MS (Thermo Trace GC Ultra/ThermoTrace ISQ, Courtaboeuf, France). Helium was used as the carrier gas at a constant flow of 1.2 mL/min. The injection port was held at 230 °C and used in the split mode (split ratio: 1/50). The transfer line was maintained at 250 °C. For CEO, the analysis was carried out on an Ultra Alloy capillary column (5% phenyl methyl polysiloxane; 30 m × 0.25 mm i.d. and 0.25 µm film thickness). The oven temperature was programmed from 50 °C to 300 °C at 15 °C/min; the final temperature was maintained for 1 min. For GEO, an Optima-Wax Plus column was used (30 m × 0.25 mm i.d. and 0.25 µm film thickness). The oven temperature was programmed from 40 °C (held 1 min) to 90 °C at 5 °C/min, then to 260 °C at 15 °C/min; this final temperature was held during 10 min. In both cases, the mass spectrometer was used in electronic ionization mode (70 eV) with a source at 200 °C and an acquisition mass range from *m*/*z* 30 to *m*/*z* 800. The relative percentages of the components were calculated from their peak areas in the total ion chromatogram (TIC) using the Excalibur 2.1 software of the device. Compounds were identified by comparison of their mass spectral fragmentation patterns with those present in the NIST 2.0f (2008) library. The most likely structures were retained.

### 3.3. Preparation of Film-Forming Dispersions (FFDs)

FFDs were prepared as described by Noshirvani et al. [29], with some modifications. Briefly, chitosan (0.2 g in 50 mL distillated water) dispersed in acidic water (0.5% *v*/*v* acetic acid) was stirred overnight (400 rpm); the pH was adjusted to 8 with NaOH 3 M. Carboxymethyl cellulose sodium salt (CMC, 0.4 g) was dissolved in distillated water (50 mL). Then, both solutions were mixed and Tween 80 (0.2 mL) was added as an emulsifier. The mixture was stirred for 15 min. After addition of oleic acid (0.3 mL), the mixture was homogenized by probe ultrasound (Bandelin Sonopuls, Berlin, Germany) for 15 min; it became turbid and milky white in the end. Glycerol was added as a plasticizer (0.3 mL; 63% *w*/*w* glycerol/polysaccharides) and the mixture was stirred again for 15 min. Then, different amounts (25, 50, 75 and 750 µL) of essential oil were added followed by sonication for 15 min. Thus, films contained 4.4, 8.8, 13.2 and 131.8% *w*/*w* (EO/Polysaccharides) for films incorporated with cinnamon EO (CEO 1, CEO 2, CEO 3 and CEO 4, respectively) and 3.5, 4.5, 10.6 and 106% *w*/*w* (EO/polysaccharides) for films incorporated with ginger EO (GEO 1, GEO 2, GEO 3 and GEO 4, respectively). FFDs were used as the coatings for sliced soft bread or for the preparation of films. In the latter case, the FFDs (50 mL for each film) were poured into Petri dishes (9 cm diameter) and dried at ambient temperature and 50% relative humidity (RH).

### 3.4. Characterization of Films

#### 3.4.1. Attenuated Total Reflectance-Fourier Transform Infrared (ATR-FTIR) Spectroscopy

The ATR-FTIR spectra of films were registered at room temperature on a Bruker VERTEX 70 instrument (Wissembourg, France) equipped with a Pike Technologies (Fitchburg, WI, USA)/Gladi ATR plate (diamond crystal) (range: 400–4000 cm^−1^, 32 scans, resolution: 4 cm^−1^).

#### 3.4.2. Scanning Electron Microscopy (SEM)

Microstructure analysis of the surfaces and cross sections of the films was carried out using the SEM technique with a tungsten source in a Quanta 200 device (FEI, Lexington, KY, USA). Samples were observed using low vacuum 50 Pa and 3 KV at a working distance of 8–10 mm.

#### 3.4.3. Differential Scanning Calorimetry (DSC)

Differential scanning calorimetry was achieved in duplicate using a DSC Q100-RCS (TA Instruments, New Castle, DE, USA). Conditioned samples (RH 55%, 25 °C, 72 h) were placed (≈5 mg) in a hermetically sealed aluminum pan and heated and cooled at a rate of 10 °C/min between 20 and 230 °C under a nitrogen flow (25 mL/min; reference: an empty pan).

#### 3.4.4. Antioxidant Activity of Films

##### Total Phenolic Content (TPC)

The total phenolic content of the films was measured using the Folin-Ciocalteu method. Briefly, strips of each film (2 cm × 1.5 cm; 70 mg) were dissolved in methanol (5 mL) for 5 days; then, extract solutions (0.05 mL) were mixed with Folin-Ciocalteu reagent (0.5 mL) for 8 min in dark, after which sodium carbonate (1 mL, 20%, *w*/*v*) and water (8.45 mL) were added to obtain a final volume of 10 mL. The mixture was stirred thoroughly and allowed to stand for 2 h at room temperature prior to an absorbance reading at 760 nm in a spectrophotometer. A calibration curve was established by plotting the concentration of gallic acid versus absorbance and the results were expressed as mg gallic acid equivalent (GAE) per gram of dried film. At least three replications were performed for each sample.

##### DPPH Radical Scavenging Activity

Briefly, strips of each film (2 cm × 1.5 cm; 70 mg) were dissolved in methanol (5 mL) for 5 days; then, extract solutions (2.0 mL) were mixed with a methanolic DPPH solution (150 μM; 2.0 mL) and stirred for 2 min in the dark, after which the mixture was maintained in darkness for 60 min at ambient temperature. The absorbance was measured against pure methanol at 517 nm and the percentage of DPPH radical scavenging activity was calculated using the following Equation (1):

DPPH scavenging activity (%) = 100 (A_sample_ − A_blank_)/A_blank_ (1)

where A_blank_ is the absorbance of the methanolic solution of DPPH and A_sample_ is the absorbance of the sample extract. At least three replications were performed for each sample.

#### 3.4.5. In Vitro Antifungal Activity of Essential Oils and Films

The antifungal properties of the films against the growth of *Penicillium digitatum* were tested via the disc diffusion method. *P. digitatum* was isolated from moldy bread and identified using morphological tests.

The fungal strain was inoculated on Potato Dextrose Agar (PDA) and incubated at 25 °C until sporulation. Then, the spores were re-suspended in physiological water with Tween 80 (0.1%, *w*/*w*) and the concentration was adjusted to 10^6^ spores/mL with physiological water using a Neubauer hemocytometer (Simax Kavalier, Prague, Czech republic). The fungal suspension (100 μL) was spread on the solidified PDA culture media and used for the determination of the minimum inhibitory concentration (MIC) of essential oils and antifungal activity of the films.

##### Determination of the MIC of Essential Oils

Appropriate amounts of essential oil were mixed in dimethylsulfoxide (DMSO) to obtain different concentrations of EOs. Sterile paper discs (diameter 4 mm) were deposited on the inoculated medium surface, then different serial dilutions of both EOs were added to the discs. The plates were sealed using Parafilm^®^ before incubation at 25 °C. Three replicates were prepared for each concentration. The lowest concentration of EO, which inhibited fungal growth, was reported as the MIC.

##### Determination of the Antifungal Activity of Films

Films were cut via a sterile punch (diameter 4 mm) and deposited on the inoculated PDA medium surface. Afterwards, the plates were sealed using Parafilm^®^ before incubation at 25 °C for 5 days. A digital ruler was used to measure the diameter of the clear zone around the films (nearest 0.01 mm) and the data were expressed as the means from three repetitions.

#### 3.4.6. Antifungal Properties of Active Materials on Bread Slices

##### Numeration after Bread Slice Coating with FFDs

Film-forming dispersions, prepared as described in Section 2.3, were used as a coating on bread slices (50 mL) using a sterilized brush. Bread slices coated by sterilized water were used as control samples. After drying under the laminar flow for 1 h, the slices were packed in polyethylene bags (30 × 17 cm); then, they were stored at 25 °C and the enumeration of yeasts and molds was conducted at the selected storage times (0, 3, 7 and 15 days). For the enumeration, bread pieces (10 g) were aseptically weighed and put into a flask containing physiological water (90 mL, NaCl 0.9% *w*/*v*). After stirring for 5 min at ambient temperature, serial dilutions (10^−1^–10^−5^) were prepared and inoculated in PDA medium. The plates were incubated at 25 °C for 5 days before yeast and mold enumeration. The results are expressed in a logarithmic scale (log cfu/g) from three repetitions.

##### Visual Observation of Packaged Bread Slices during Storage

Bread slices were sandwiched between two pieces of films and packaged in polyethylene bags (30 cm × 17 cm). Then, they were kept at 25 °C until visual observation of fungal growth. Three replicates were prepared for each sample. Bread slices without any film were prepared as a control.

### 3.5. Statistical Analysis

Statistics on a completely randomized design were performed with the analysis of variance (ANOVA) procedure in SPSS (version 16, SPSS Inc., Chicago, IL, USA) software. Duncan’s multiple range test (*p* < 0.05) was used to detect differences among the mean values of the films’ properties.

## 4. Conclusions

Active films based on essential oils were successfully prepared from carboxymethylcellulose and chitosan emulsified with oleic acid. Films containing cinnamon or ginger essential oils (CEO and GEO, respectively) showed antioxidant properties and antifungal activities against a strain of *Penicillium digitatum*, with CEO consistently outperforming GEO. Soft bread slices wrapped in films containing the highest concentrations of EOs remained yeast and mold free from 7 to 28 days for GEO and up to 60 days for CEO. These CMC-CH-OL-based films with added CEO or GEO appear promising for the preservation of sliced soft bread (considered particularly sensitive to contamination), potentially improving food safety and quality. Clearly, further studies are needed to assess the effectiveness of this material against fungal contamination on other types of bread, such as loaves. This study is aimed more at testing the feasibility and potential efficacy of these active materials on a model loaf. In addition, further studies, including evaluations by consumer panels, are now needed to assess the sensory and organoleptic properties of bread protected by these films.

## Figures and Tables

**Table 1:** In vitro antifungal properties against *P. digitatum* of films with cinnamon or ginger essential oils after 5 days of incubation at 25 °C and MIC values. Data represent means and standard deviations from three repetitions (different superscript values are significantly different (*p* < 0.05).

Films	CEO 1	CEO 2	CEO 3	CEO 4	GEO 1	GEO 2	GEO 3	GEO 4
Inhibition zone diameter (mm)	11.5 ± 0.4 ^d^	17.9 ± 1.3 ^c^	20.9 ± 1.8 ^b^	69.7 ± 2.3 ^a^	2.9 ± 0.5 ^f^	3.7 ± 0.3 ^f^	7.3 ± 0.4 ^e^	22.8 ± 0.3 ^b^
Inhibition (%)	13	20	23	83	4	5	10	26
MIC value (mL/mL)		0.03 ± 0.02					4.00 ± 1.03	

**Table 2:** Evaluation of bread yeast and mold contamination during incubation at 25 °C. Numeration over time (log CFU/g). Data represent means and standard deviations from three repetitions.

Storage Days	Control	CMC-CH	CMC-CH-OL	CEO 1	CEO 4	GEO 1	GEO 4
0	2.35 ± 0.02 ^d^	2.22 ± 0.02 ^d^	1.92 ± 0.02 ^c^	0.0 ± 0.0 ^d^	0.0 ± 0.0 ^a^	2.03 ± 0.01 ^d^	1.39 ± 0.02 ^d^
3	5.00 ± 0.03 ^c^	4.57 ± 0.03 ^c^	4.33 ± 0.03 ^b^	0.5 ± 0.01 ^c^	0.0 ± 0.0 ^a^	4.08 ± 0.02 ^c^	2.00 ± 0.02 ^c^
7	6.26 ± 0.03 ^b^	5.56 ± 0.03 ^b^	6.11 ± 0.03 ^a^	2.25 ± 0.02 ^b^	0.0 ± 0.0 ^a^	5.36 ± 0.03 ^b^	3.39 ± 0.03 ^b^
15	7.46 ± 0.04 ^a^	7.05 ± 0.04 ^a^	6.71 ± 0.04 ^a^	4.45 ± 0.04 ^a^	0.0 ± 0.0 ^a^	6.53 ± 0.03 ^a^	6.00 ± 0.04 ^a^

Columns with the same letter are not significantly different (*p* < 0.05).

**Table 3:** Visual observation of yeasts and molds in bread sandwiched between different films and stored at 25 °C for a maximum of 35 days. Data with the same letter are not considered as different.

Type of Packaging	Growth Delay (Days)	Intensity of Fungal Growth *			
		Day 7	Day 15	Day 28	Day 35
CMC-CH-OL	7.0 ^e^	+	++	+++	+++
CMC-CH-OL-CEO 1	14.5 ^d^	-	+	+	++
CMC-CH-OL-CEO 2	20.5 ^b^	-	-	+	+
CMC-CH-OL-CEO 3	NG **^f^	-	-	-	-
CMC-CH-OL-CEO 4	NG **^f^	-	-	-	-
CMC-CH-OL-GEO 1	14.5 ^d^	-	+	+	++
CMC-CH-OL-GEO 2	14.0 ^d^	-	+	+	++
CMC-CH-OL-GEO 3	18.0 ^c^	-	-	+	++
CMC-CH-OL-GEO 4	28.0 ^a^	-	-	+	+

* - No fungal growth; + fungal growth lower than 25% of slice surface; ++ fungal growth between 25 to 50% of slice surface; +++ fungal growth around 75% of slice surface, ** NG: No fungal growth within 2 months.

## Data Availability

The data presented in this study are available on request from the corresponding author.

## Supplementary Materials

The following supporting information can be downloaded at: https://www.mdpi.com/article/10.3390/molecules29194664/s1, Supplementary Part A: Calculation of the activation energy of cinnamon and ginger essential oils; Supplementary Part B: Figure S1: DSC thermograms of pure essential oils (CEO and GEO) and thermograms of CMC-CH-OL films loaded with CEO 4 and GEO 4; Figure S2: TGA curves of cinnamon and ginger essential oils at different heating rates; Figure S3: Values of total phenolic contents (A) and DPPH scavenging activity (B) of the different films; Table S1: Moisture content and water activity. References [61,62,63] are cited in the Supplementary Materials.
