# Supplementary material for: Active Polysaccharide-Based Films Incorporated with Essential Oils for Extending the Shelf Life of Sliced Soft Bread
Source: Molecules. 2024 Sep 30;29(19):4664. doi: 10.3390/molecules29194664 (PMC11477974; doi:10.3390/molecules29194664)
Supplement: Supplementary file 1 [file molecules-29-04664-s001.zip › molecules-3117172-supplementary.pdf]

Active Polysaccharide-Based Films Incorporated with Essential Oils for Extending the Shelf Life of Sliced Soft Bread

Nooshin Noshirvani[a,b], Cédric Le Coz[a], Christian Gardrat[a], Babak Ghanbarzadeh[c], Véronique Coma[a*]

[a] Laboratoire de Chimie des Polymères Organiques, Université de Bordeaux, CNRS, Bordeaux INP, UMR 5629, 16 Avenue Pey-Berland, F-33600 Pessac, France
[b] Department of Food Science and Technology, Tuyserkan Faculty of Engineering & Natural Resources, Bu-Ali Sina University, Hamedan, Iran
[c]Department of Food Science and Technology, Faculty of Agriculture, University of Tabriz, Tabriz, Iran

SUPPLEMENTARY DATA

PART A
Calculation of the activation energy of cinnamon and ginger essential oils. Page 2

PART B
Figure S1. DSC thermograms of pure essential oils (CEO and GEO) and thermograms of CMC-CH-OL films loaded with CEO 4 and GEO 4 Page 4

Figure S2. TGA curves of cinnamon and ginger essential oils at different heating rates Page 4

Figure S3. Values of total phenolic contents (**A**) and DPPH scavenging activity (**B**) of the different films Page 5

Table S1. Moisture content and water activity Page 6

PART A Calculation of the activation energy of cinnamon and ginger essential oils

Thermogravimetric measurements (TG) of essential oils (around 12 mg) were performed on a TA Instruments Q500 (USA) from room temperature to 950 °C under a nitrogen flow rate of 60 mL min^{-1}. Kinetic studies were conducted using data obtained from the TG analyses, which were measured at varied heating rates of 2, 5, 10 and 20 °C min^{-1}.

Decomposition kinetics from TG data are theoretically based on the isoconversional method which states that the reaction rate at a constant extent of conversion is only dependent on temperature. Thus, the temperature dependence of the isoconversional rate can be used to evaluate the activation energy (E_a) of essential oil degradation without determining the reaction model. The decomposition kinetics of EOs can be described using a general form of the equation

$$d\alpha/dt = A\, e^{-Ea/RT} (1 - \alpha)^{n} \qquad (1)$$

where α is the fraction of the sample degraded, representing the reaction rate, A is the pre-exponential factor, E_a is the activation energy, R is the gas constant, T is the absolute temperature and n is the reaction order.

Non-isothermal rate of degradation using TG data could be described by the following expression:

$$d\alpha/dT = (d\alpha/dt)\,(dt/dT) \qquad (2)$$

where dα/dT is the non-isothermal rate; dT/dt is the rate of heating which could be described as variable β; dα/dt is the isothermal reaction rate. Thus, non-isothermal rate equation of decomposition could be derived as follows:

$$d\alpha/dT = A/\beta\, e^{-Ea/RT} (1 - \alpha)^{n} \qquad (3)$$

For the decomposition process, isoconversion kinetic methods are useful to analyze the values of E_a which is based on integral calculations derived from Equation (3) without evaluating the particular form of reaction model.

The Flynn–Wall–Ozawa (FWO) and Kissinger–Akahira–Sunose (KAS) model equations were used to determine E_a for CEO and GEO according to E698 and E2890 ASTM norms. Four runs with different heating rates were used to obtain TG data.

For the FWO model, the following equation was used:

$$\ln \beta = \text{constant} - 1.052\, E_a/RT_{max} \qquad (4)$$

E_a can be obtained from the linear correlation of ln β versus $1/T_{max}$ for the four heating rates.

For the KAS model, the following equation was used:

$$\ln (\beta/T_{max}^{2}) = \text{constant} - (E_a/RT_{max}) \qquad (5)$$

$\ln(\beta/T^2)$ versus $1/T_{max}$ data points obtained from the four heating rates were plotted and fitted to a straight line and the slope was used to measure the activation energy.

For both models, T_{max} corresponds to the temperature at which the rate of degradation is the highest.

The best lines and the standard deviation of the regression coefficients were calculated using the methods of least squares. The activation energy of each essential oil with both models are indicated on the following figures.

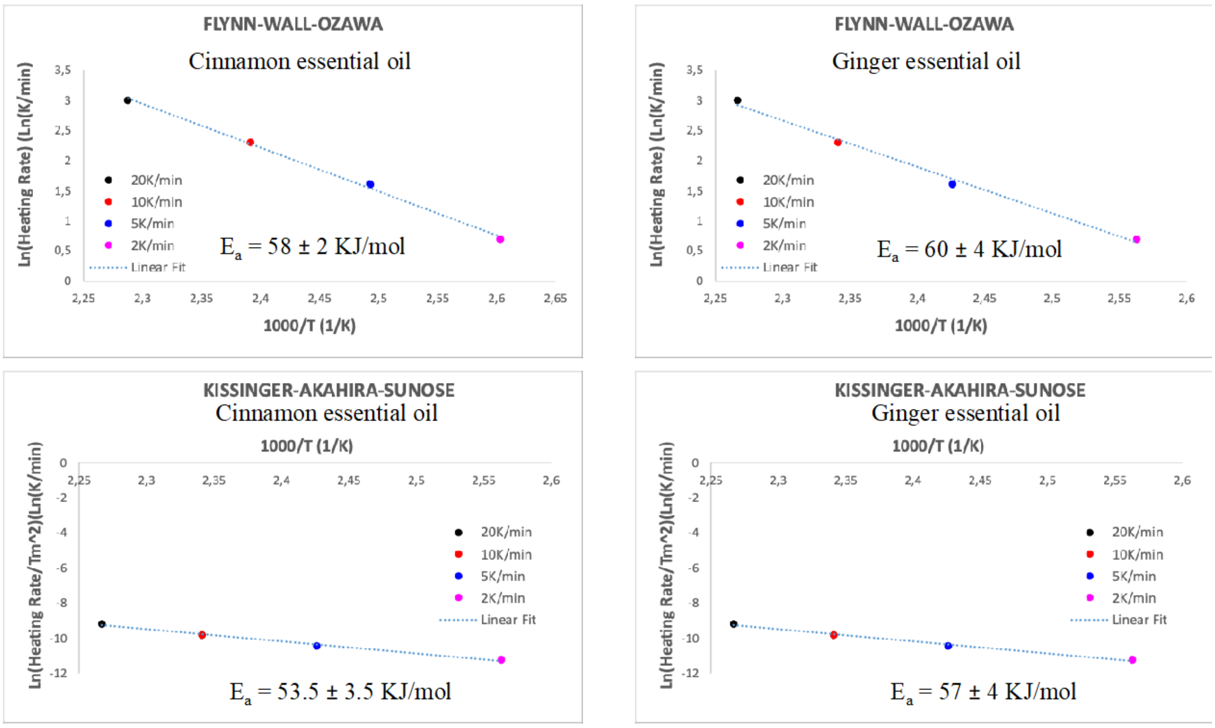

Figures: Calculated activation energies of commercial cinnamon and ginger essential oils with their standard deviations

Literature

[61] Simon, P. 2004). Isoconversional methods. *Journal of Thermal Analysis and Calorimetry*, *76*, 123–132. doi:10.1023/B:JTAN.0000027811.80036.6c).
[62] *ASTM E698-18*; Standard Test Method for Kinetic Parameters for Thermally Unstable Materials Using Differential Scanning Calorimetry and the Flynn/Wall/Ozawa Method. ASTM International: West Conshohocken, PA, USA, 2023.
[63] *ASTM E2890-12*; Test Method for Kinetic Parameters for Thermally Unstable Materials by Differential Scanning Calorimetry Using the Kissinger and Farjas Methods E2890 Test method for kinetic parameters for thermally unstable materials by differential scanning calorimetry using the Kissinger and Farjas methods. ASTM International: West Conshohocken, PA, USA, 2015.

PART B:

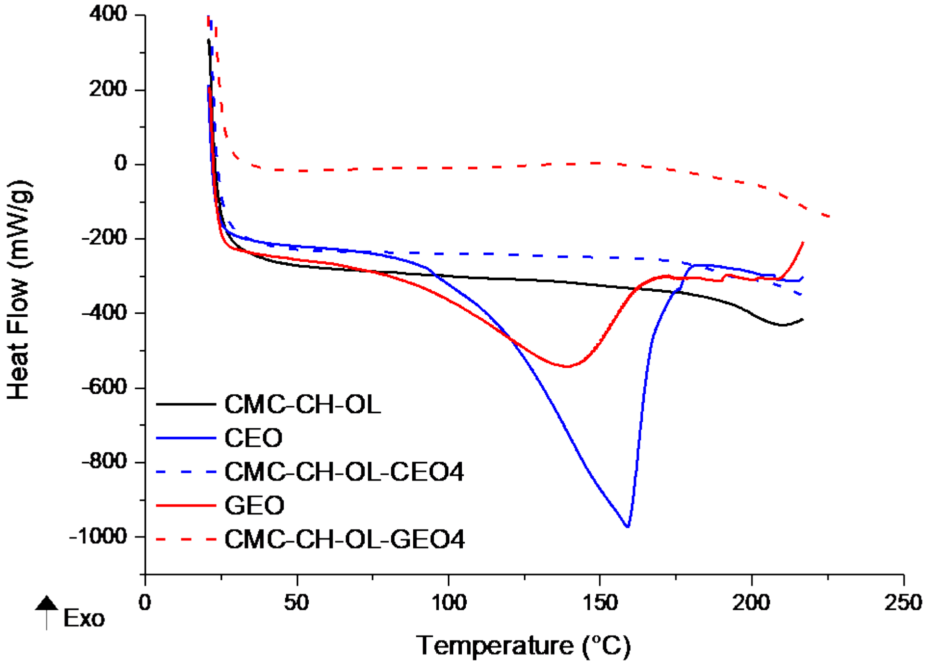

Figure S1. DSC thermograms of pure essential oils (CEO and GEO) and thermograms of CMC-CH-OL films loaded with CEO 4 and GEO 4

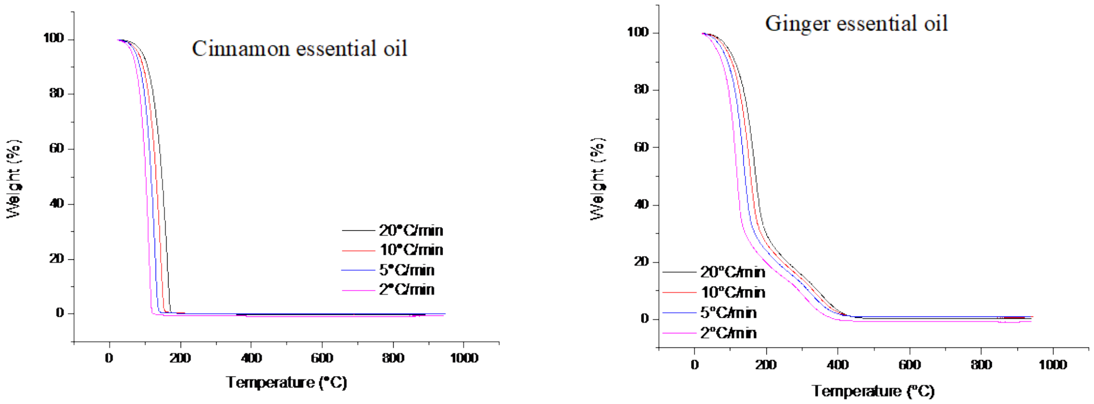

Figure S2. TGA curves of cinnamon and ginger essential oils at different heating rates

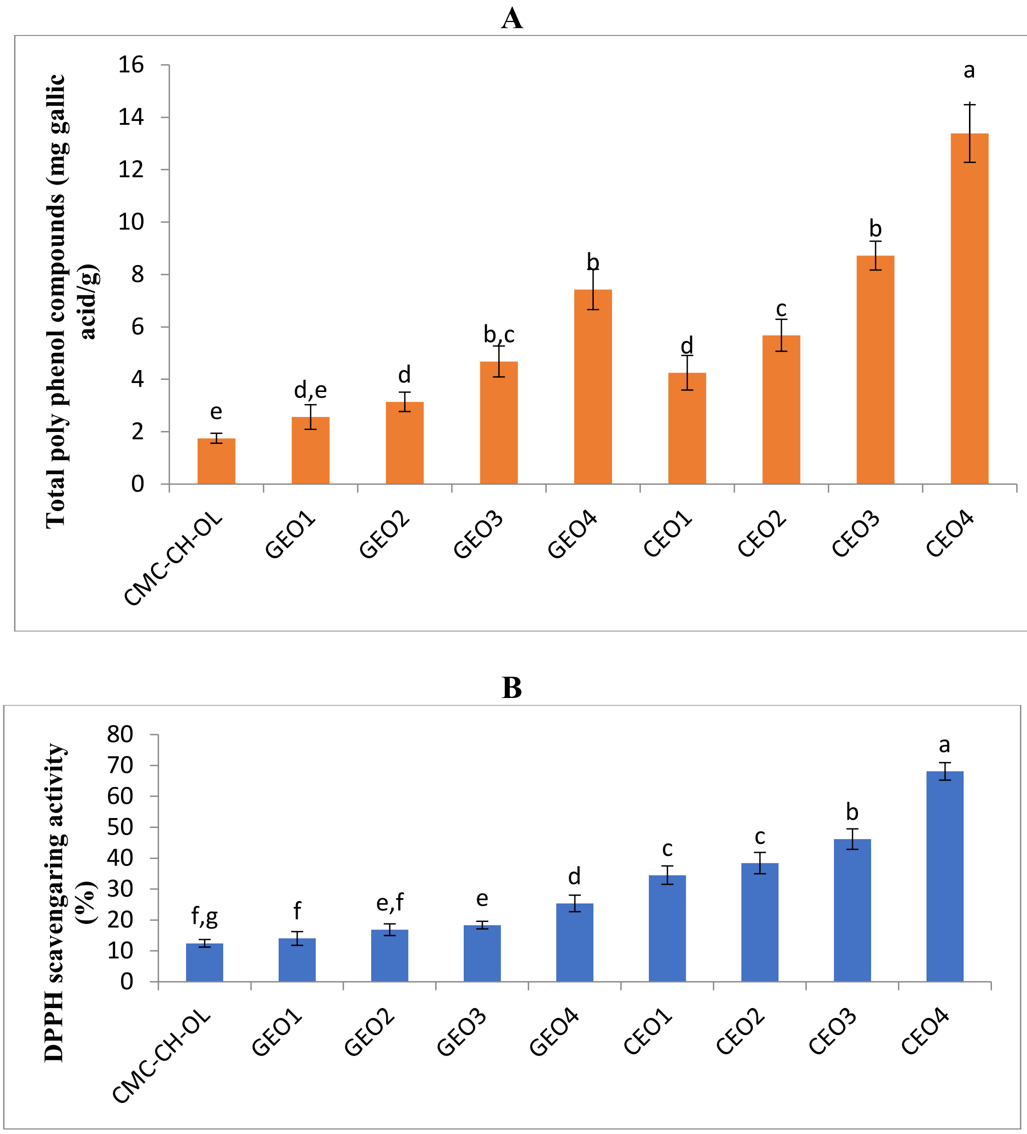

Figure S3. Values of total phenolic contents (**A**) and DPPH scavenging activity (**B**) of the different films

Table S1. Moisture content and water activity

	Moisture content (%)	Water activity a_w
Control	21.2±2.0^{c}	0.86±0.01^{c}
CMC-CH	31.2±2.5^{b}	0.89±0.02^{b}
CMC-CH-OL	34.5±3.2^{a,b}	0.91±0.01^{a,b}
CEO 3	35.0±1.8^{a}	0.93±0.02^{a}
CEO 4	37.1±3.1^{a}	0.93±0.01^{a}
GEO 3	35.2±2.9^{a}	0.93±0.02^{a}
GEO 4	36.3±1.7^{a}	0.93±0.08^{a}

Moisture content of coated bread slices with different formulations, was evaluated at 110°C based on initial weight and constant final weight of bread slices. After drying, the samples were cooled in a desiccator at ambient temperature.
Water activity was determined at 25°C using an a_w meter (Aqua Lab Series 3 TE, Decagon Deuices Inc, Pullman, USA). About 5.0 g of the ground breads were used for each test.
Three replications were done for each sample.
